# MAP Kinase Phosphatase 2 Regulates Macrophage-Adipocyte Interaction

**DOI:** 10.1371/journal.pone.0120755

**Published:** 2015-03-27

**Authors:** Huipeng Jiao, Peng Tang, Yongliang Zhang

**Affiliations:** 1 Department of Microbiology, Yong Loo Lin School of Medicine, National University of Singapore, Singapore, Singapore; 2 Immunology Programme, the Life Science Institute, National University of Singapore, Singapore, Singapore; Warren Alpert Medical School of Brown University, UNITED STATES

## Abstract

**Objective:**

Inflammation is critical for the development of obesity-associated metabolic disorders. This study aims to investigate the role of mitogen-activated protein kinase phosphatase 2 (MKP-2) in inflammation during macrophage-adipocyte interaction.

**Methods:**

White adipose tissues (WAT) from mice either on a high-fat diet (HFD) or normal chow (NC) were isolated to examine the expression of MKP-2. Murine macrophage cell line RAW264.7 stably expressing MKP-2 was used to study the regulation of MKP-2 in macrophages in response to saturated free fatty acid (FFA) and its role in macrophage M1/M2 activation. Macrophage-adipocyte co-culture system was employed to investigate the role of MKP-2 in regulating inflammation during adipocyte-macrophage interaction. c-Jun N-terminal kinase (JNK)- and p38-specific inhibitors were used to examine the mechanisms by which MKP-2 regulates macrophage activation and macrophage-adipocytes interaction.

**Results:**

HFD changed the expression of MKP-2 in WAT, and MKP-2 was highly expressed in the stromal vascular cells (SVCs). MKP-2 inhibited the production of proinflammatory cytokines in response to FFA stimulation in macrophages. MKP-2 inhibited macrophage M1 activation through JNK and p38. In addition, overexpression of MKP-2 in macrophages suppressed inflammation during macrophage-adipocyte interaction.

**Conclusion:**

MKP-2 is a negative regulator of macrophage M1 activation through JNK and p38 and inhibits inflammation during macrophage-adipocyte interaction.

## Introduction

Obesity—a rapidly emerging major public health issue worldwide—is associated with an increased risk of insulin resistance and type 2 diabetes (T2D) [1]. Obesity-associated inflammation in adipose tissue is critical in the initiation and progression of systemic insulin resistance [2]. Generally, expansion of adipose tissue in obesity leads to increased macrophage infiltration and inflammation with enhanced production of proinflammatory cytokines such as tumor necrosis factor α (TNF-α) and interleukin 6 (IL-6). This is accompanied by an increased release of free fatty acids (FFAs) and dysregulated secretion of adipocyte- and macrophage-derived factors, including leptin, adiponectin, and resistin [3,4]. These mediators (collectively known as adipokines) can act in a paracrine or autocrine fashion to further exacerbate adipose tissue inflammation and reduce insulin sensitivity [5].

In mice, macrophages are the major immune cells infiltrated in adipose tissue in response to high-fat diet (HFD) [6]. Adipose tissue macrophages (ATMs) are a prominent source of proinflammatory cytokines such as TNF-α, IL-6, and IL-1β that can block insulin action [4,7]. During HFD-induced progressive obesity, ATMs undergo a phenotypic switch from an anti-inflammatory M2 polarization state to a proinflammatory M1 polarization state [7]. M1 or “classically activated” macrophages promote insulin resistance, whereas M2 or “alternatively activated” macrophages are protective against the development of insulin resistance. M1 macrophage activation can be induced *in vitro* by proinflammatory mediators such as interferon (IFN)-γ and lipopolysaccharides (LPS) [8], while M2 macrophages can be induced by exposure to IL-4 and IL-13 [7,9].

Mitogen-activated protein kinase (MAPK) phosphatases (MKPs) or dual specificity phosphatases (DUSPs) are major negative regulators of MAPKs [10]. They inactivate MAPKs through dephosphorylation of threonine and/or tyrosine residues essential for the activation of MAPKs. Members of MKP family have been shown to play diverse roles in metabolism. For instance, MKP-4 was reported to inhibit insulin-stimulated adipogenesis and glucose uptake in adipocytes [11]. In addition, it played a protective role in the development of stress-induced insulin resistance [12]. Wu et al showed that mice lacking MKP-1 were resistant to diet-induced obesity due to enhanced energy expenditure [13]. More recently, MKP-3 was shown to promote hepatic gluconeogenesis by dephosphorylation of forkhead transcription factor FOXO1 [14]. MKP-2 is a 42-kDa inducible phosphatase known to be upregulated in response to growth factors, phorbol 12-myristate 13-acetate (PMA), oxidative stress, and UV light as well as LPS [15]. Interestingly, one study on MKP-2 showed that it is a negative regulator of c-Jun N-terminal kinase (JNK) and p38 in macrophages and that it inhibits the expression of proinflammatory cytokines in response to LPS [16]. Cornell et al, on the other hand, showed that MKP-2 is an extracellular signal-regulated kinase (ERK) phosphatase and in response to LPS, inhibits MKP-1 expression through ERK to enhance the expression of inflammatory cytokines such as TNF-α in macrophages [15]. In addition to such controversies on its substrates, the role of MKP-2 in macrophage M1/M2 activation and its function in ATMs are not well studied. In this study, we showed that MKP-2 inhibits inflammatory activation of macrophages and macrophage-mediated inflammation during the macrophage-adipocyte interaction through JNK and p38.

## Materials and Methods

### Animal experiment

Animal experiments were approved by the Institutional Animal Care and Use Committee of National University of Singapore. 5–6 weeks old male C57BL/6 mice (4–5 mice per group) were fed with a chow diet (NC) or a high-fat diet (HFD; TD03584, Harlan) (35.2% fat, 20.4% protein, and 36.1% carbohydrate by weight) for 8 weeks. The mice were sacrificed by CO_2_ gas asphyxiation without fasting. Adipose tissue was isolated and total RNA extracted for cDNA synthesis. To isolate stromal vascular cells (SVCs), adipose tissue was minced into fine pieces immediately after CO_2_ asphyxiation. Minced samples were digested in HEPES-buffered DMEM supplemented with 2.5% bovine serum albumin (BSA) and 40μg/mL collagenase at 37°C on an orbital shaker (200rpm) for 45–60 min. The digested samples were passed through a sterile 100μm nylon mesh and the suspension was placed on ice for 20min followed by centrifugation at 1000rpm for 5min. The floating adipocytes and the pelleted SVCs were separated for RNA extraction. The red blood cells within SVCs were lysed in ACK (Ammonium-Chloride-Potassium) Lysing Buffer (Lonza).

### Cell culture

RAW264.7 (ATCC) macrophage cell line was maintained in RPMI1640 medium (Invitrogen) containing 10% heat-inactivated fetal bovine serum (FBS) (Invitrogen) and 1% Pen/Strep (Invitrogen). 3T3-L1 preadipocytes, a preadipocyte cell line commonly employed as a preadipose cell model [17], were maintained in DMEM medium (Invitrogen) containing 10% bovine serum (Invitrogen), 1% Pen/Strep (Invitrogen), and 1mM sodium pyruvate (Invitrogen). Cells were incubated at 37°C in a humidified 5% CO_2_/95% air atmosphere. Differentiation of 3T3-L1 preadipocytes into mature adipocytes was performed using insulin (Sigma), dexamethasone (Sigma), and 3-isobutyl-1-methly-xanthine (Sigma) for 8 days as described [18]. Macrophage M1 phenotype was induced by culturing RAW264.7 in presence of 20ng/mL of mouse recombinant IFN-γ (BD Pharmingen) for 12h followed by 100ng/mL LPS (Sigma) stimulation for another 12h. M2 activation of macrophages was induced by either 20ng/mL IL-4 (Biolegend) or 20ng/mL IL-13 (Biolegend) for 12h. Palmitate-BSA (Sigma) complex preparation was performed as described previously [19]. Briefly, palmitate was dissolved in 95% ethanol at 60°C and mixed with pre-warmed 10% FFA-free BSA (Sigma), yielding a stock concentration of 7.5mM.

### Dual luciferase reporter assay

2 × 10^5^ RAW264.7 cells were co-transfected with 50 ng AP-1 reporter construct, 50ng pcDNA3.1-MKP-2 together with 10 ng of pRL-null plasmid using Lipofectamine LTX reagent (Invitrogen). Equal amounts of pcDNA3.1 empty vector were transfected as control. Reporter activity was determined with Promega Dual Luciferase Assay System (Promega). Firefly luciferase values were normalized for transfection efficiency by means of the *Renilla* luciferase activity that is constitutively expressed by pRL-null.

### Generation of MKP-2 overexpressing RAW264.7 cells

pcDNA3.1-MKP-2 or pcDNA3.1 empty vector was transfected into RAW264.7 cells using Lipofectamine LTX (Invitrogen). Transfected cells were cultured in RPMI complete medium containing 500μg/mL G418 (Clontech), and the medium was changed every 2 days for 14 days. Cell colonies were picked after selection. MKP-2 mRNA and protein expression were measured to determine MKP-2 expression. Selected cells were maintained in complete medium in the presence of 100ng/mL G418.

### Quantitative real-time polymerase chain reaction

Total RNA was extracted from cultured cells using TRIzol (Invitrogen) and used for cDNA synthesis using ImProm-II Reverse Transcription System (Promega). Quantitative real-time PCR (qRT-PCR) was performed with an Applied system 7900 Detection System using Fast SYBR Master Mix (Applied Biosystems, B.V). The following mouse primers were used: forward primer 5’-GCAGTGCCTACCATGCTG-3’ and reverse primer 5’-ATGGCTTCCATGAACCAGGAG-3’ for *Mkp-2*; forward primer 5’-CTTGCAGATGAAGCCTTTGAAGA-3’ and reverse primer 5’-GGAACGCACCTTTCTGGACA-3’ for *Interleukin-12 p40 (Il12p40)*; forward primer 5’-CTCCAAGCCAAAGTCCTTAGAG-3’ and reverse primer 5’-AGGAGCTGTCATTAGGGACATC-3’ for *Arginase1* (*Arg1)*; forward primer 5’-AGAAGGGAGTTTCAAACCTGGT-3’ and reverse primer 5’-GTCTTGCTCATGTGTGTAAGTGA-3’ for *Chitinase 3-like 3* (*Chi3l3)*; forward primer 5’-TGAGAAAGGCTTTAAGAACTGGG-3’ and reverse primer 5’-GACCACCTGTAGTGATGTGGG-3’ for *Macrophage galactose N-acetyl-galactosamine specific lectin 1* (*Mgl1)*; forward primer 5’-GCTCTTACTGACTGGCATGAG-3’ and reverse primer 5’-CGCAGCTCTAGGAGCATGTG-3’ for *Interleukin-10* (*Il-10)*; forward primer 5’-GACAACTTTGGCATTGTG-3’ and reverse primer 5’-ATGCAGGGATGATGTTCTG-3’ for *Glyceraldehyde 3-phosphate dehydrogenase (Gapdh)*. Levels of mRNA were calculated using 2^–ΔΔ Ct^ method [20] and normalized to those of *Gapdh* mRNA.

### Co-culture of adipocytes and macrophages

Adipocyte-macrophage co-culture was performed in a contact system. Briefly, the differentiated 3T3-L1 adipocytes were cultured in a six-well plate and 1x 10^5^ RAW264.7 cells were plated onto 3T3-L1 adipocytes. The cells were co-cultured for 24-h followed by 24-h palmitate stimulation. Culture supernatants from the co-culture were harvested. Supernatants of separately cultured adipocytes and macrophages, numbers of which were equal to those in the contact system, were used as control. All the cytokines production was normalized to the total protein of the cell lysates.

### Enzyme-linked immunosorbent assay (ELISA)

The concentrations of IL-6 and TNF-α in culture supernatants were determined by IL-6 and TNF-α ELISA kits (BD Pharmingen). Monocyte chemotactic protein 1 (MCP-1) concentration was determined using an ELISA kit from eBioscience.

### Western blot

Whole-cell lysates were separated by 10% sodium dodecyl sulfate polyacrylamide gel electrophoresis (SDS-PAGE), and western blotting was performed using antibodies against MAPK (Cell signaling), MKP-2 (Santa cruz), and β-actin (Cell signaling). Immunoblots were developed with enhanced chemiluminescence (ECL) donkey anti-rabbit IgG linked to horseradish peroxidase secondary antibodies (GE Healthcare) and SuperSignal West Dura Chemiluminescent Substrate (Thermo scientific). The blots were exposed to Amersham Hyperfilm ECL and MP Autoradiography Films (GE Healthcare). The intensity of the indicated bands was measured using ImageJ software.

### JNK and p38 inhibition

RAW264.7 cells were pretreated with either JNK inhibitor SP600125 (20μM) (Sigma) or p38 inhibitor SB23580 (20μM) (Sigma) for 1h. After pretreatment, the cells were stimulated with IFNγ plus LPS for 0h, 0.5h, 1h and 3h to examine the activation of JNK or the phosphorylation of ATF2 by western blot using antibody against phosphor (p)-JNK, p-ATF2, JNK, and ATF2. To examine M1, M2 or inflammatory gene expression, after pretreatment with the respective inhibitor, cells were stimulated with M1 activators in the presence of SP600125 (20μM) or SB23580 (20μM) for indicated times or co-cultured with 3T3-L1 adipocytes for 24 h. DMSO was used as a vehicle control.

### Statistical analysis

Data were expressed as the mean±standard error of the mean (SEM). Statistical analysis was performed using two-tailed student’s unpaired *t*-test. A *p-*value <0.05 was considered to be statistically significant.

## Results

### Nutrition conditions modulate the expression of MKP-2 in adipose tissue

**Fig 1:**
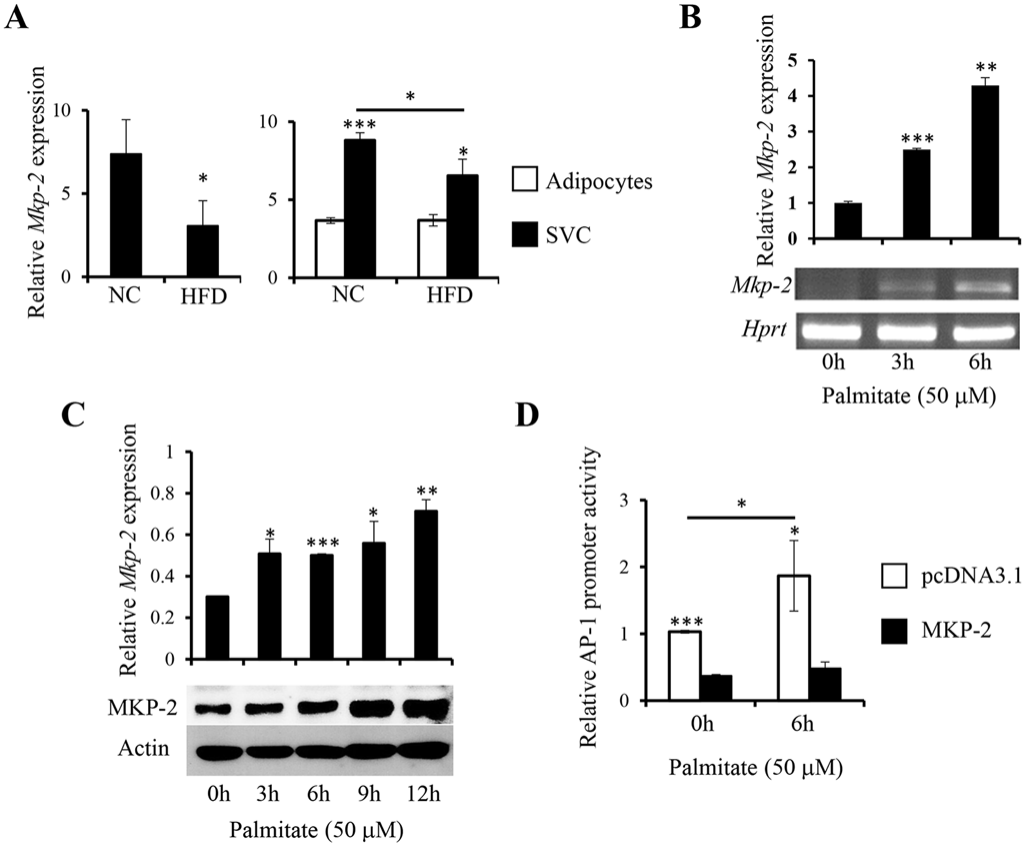
Modulation of MKP-2 expression in adipose tissue by HFD feeding and in macrophages by FFA stimulation. *(A)* WAT was isolated from mice fed with either a chow diet (NC) or a high-fat diet (HFD) for 8 week (4–5 mice per group). SVCs and adipocytes were isolated from the WAT to examine the expression of *Mkp-2* by quantitative real-time PCR (qRT-PCR). *(B) Mkp-2* expression was determined by qRT-PCR in RAW264.7 in response to 50μM palmitate stimulation. *(C)* MKP-2 protein expression was examined by western blot analysis in RAW264.7 in response to 50μM palmitate stimulation. The level of β-actin was examined as loading control. The intensity of the MKP-2 bands on the blots was quantified by ImageJ showing mean ± SEM from three independent experiments normalized to β-actin. *(D)* RAW264.7 cells were transfected with pcDNA3.1 or pcDNA3.1-MKP-2 plasmids together with AP-1 reporter plasmid and *Renilla* luciferase reporter vector. Dual luciferase assay was performed to assess AP-1 activity in response to 50μM FFA stimulation for 6h. The data shown are representative of three independent experiments with similar results. Data are presented as mean ± SEM. *, p<0.05; **, p<0.01; ***, p<0.001.

Adipose tissue dysfunction is a primary defect in obesity and obesity-associated metabolic disorders such as T2D [21]. To examine the possible role of MKP-2 in adipose tissue in obesity, 5–6 weeks old male C57BL/6J mice were fed with either HFD or NC for 8 weeks and the expression of MKP-2 in WAT was examined. We found a significant decrease in the expression of MKP-2 in WAT from HFD-fed mice compared to that from NC-fed mice (Fig. 1A). Furthermore, the expression of MKP-2 was significantly higher in SVCs isolated from WAT as compared to that in adipocytes from NC- or HFD-fed mice (Fig. 1A). Although the expression of MKP-2 in adipocytes was comparable between NC- and HFD-fed mice, there was a significant decrease in MKP-2 expression in SVCs from HFD-fed mice compared with SVCs from NC-fed mice (Fig. 1A). These results suggest a possible role of MKP-2 in WAT inflammation.

### FFA increases MKP-2 expression in macrophages

In obesity, increased release of FFAs results in the activation of inflammatory signaling pathways, recruitment of macrophages, and the production of inflammatory mediators, which contribute to the development of metabolic disorders such as insulin resistance [3]. Macrophages are the major source of inflammatory cytokines in WAT [4]. To understand the regulation of inflammatory cytokine expression in macrophages by MKP-2 in obesity, we assessed the expression of MKP-2 in macrophages in response to palmitate, one of the most abundant saturated FFAs in plasma [22]. Murine macrophage cells, RAW264.7, were stimulated with palmitate for various time periods to determine the expression of MKP-2. As shown in Fig. 1B, MKP-2 mRNA expression was significantly increased at 3 and 6h after FFA stimulation compared with that in cells without stimulation. In line with the increased mRNA expression, we detected increased MKP-2 protein expression in RAW264.7 cells after palmitate stimulation (Fig. 1C). Thus, MKP-2 expression was increased upon FFA stimulation, suggesting the possible regulatory function of MKP-2 in FFA-induced inflammation in macrophages.

### MKP-2 inhibits TNF-α expression and JNK activation in macrophages upon FFA stimulation

**Fig 2:**
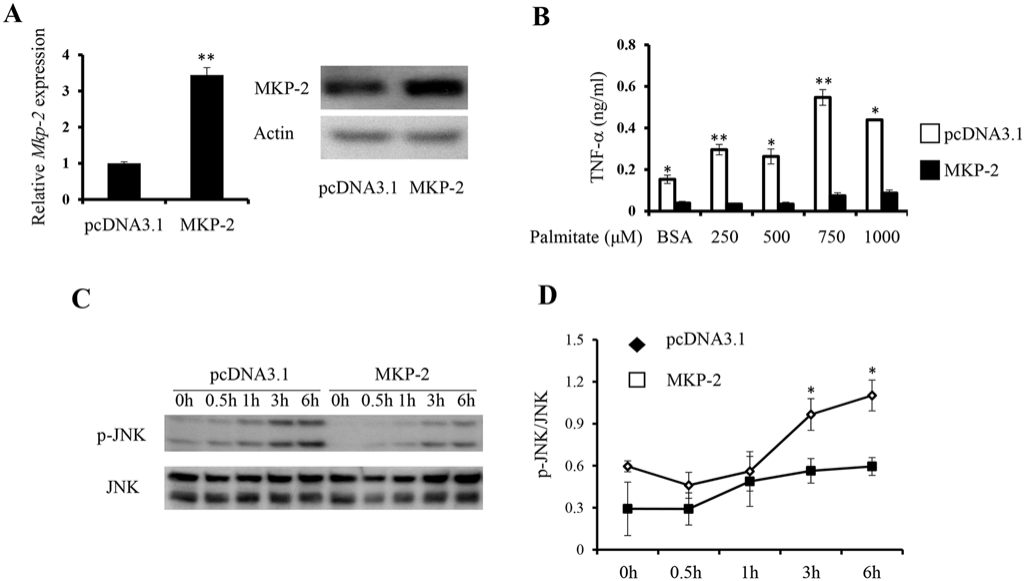
Overexpression of MKP-2 inhibits TNF-α expression and JNK activation in macrophages in response to FFA. *(A)* MKP-2 expression in MKP-2 stably transfected RAW264.7 was examined by qRT-PCR and western blot. *(B)* Control and MKP-2 overexpressing RAW264.7 cells were stimulated with indicated concentrations of palmitate for 24 h, and culture supernatants were harvested to measure TNF-α production by ELISA. *(C)* Control and MKP-2 overexpressing RAW264.7 cells were stimulated with 750μM palmitate for the indicated time points and cell lysates were prepared to examine JNK expression and activation. *(D)* The intensity of the p-JNK bands on the blots was quantified by ImageJ showing mean ± SEM from three independent experiments normalized to total JNK. Data are presented as mean ± SEM. *, p<0.05; **, p<0.01; ***, p<0.001.

To further understand the role of MKP-2 in regulating FFA-mediated inflammatory cytokine expression in macrophages, we transfected promoter construct of AP1, a major target of MAPK pathways [23], with either a full length mouse MKP-2 cDNA construct or a vector control into RAW264.7 cells. Dual luciferase assay was performed to assess AP-1 promoter activity in response to FFA. We observed that palmitate significantly increased AP-1 promoter activity in macrophages, while overexpression of MKP-2 significantly inhibited AP-1 promoter activity with or without palmitate stimulation (Fig. 1D). To elucidate the regulatory role of MKP-2 in the expression of inflammatory cytokines in response to FFA, we generated RAW264.7 cells stably expressing mouse MKP-2. qRT-PCR and western blot analysis of MKP-2 expression confirmed the overexpression of MKP-2 in selected cells at both mRNA and protein levels (Fig. 2A).

Adipose tissue-derived TNF-α, mainly from ATMs, is a MAP kinase target gene and has been shown to be a major player in obesity-induced insulin resistance and T2D [2,24,25]. To examine the role of MKP-2 in the regulation of TNF-α expression in macrophages in response to FFA, we stimulated MKP-2 overexpressing macrophages and control cells with different concentrations of palmitate. Consistent with a previous study [19], palmitate induced TNF-α production in macrophages in a dose-dependent manner. Interestingly, overexpression of MKP-2 significantly inhibited TNF-α production in response to FFA stimulation (Fig. 2B).

The substrates of MKP-2 and its role in regulating cytokines expression in macrophages are controversial [15]. To further study the mechanism by which MKP-2 regulates TNF-α expression in macrophages in response to FFA, MKP-2 overexpressing macrophages and control cells were stimulated with palmitate to examine MAP kinase activation. We found that JNK activation was increased at 3 and 6h upon FFA stimulation (Fig. 2C). Overexpression of MKP-2 significantly decreased the activation of JNK in response to palmitate (Fig. 2C). However, p38 activity could not be detected under the same condition. Taken together, the results indicate that upon FFA stimulation, MKP-2 regulates TNF-α expression in macrophages possibly through JNK.

### MKP-2 expression in macrophages suppresses inflammatory cytokine production during adipocyte-macrophage interaction

**Fig 3:**
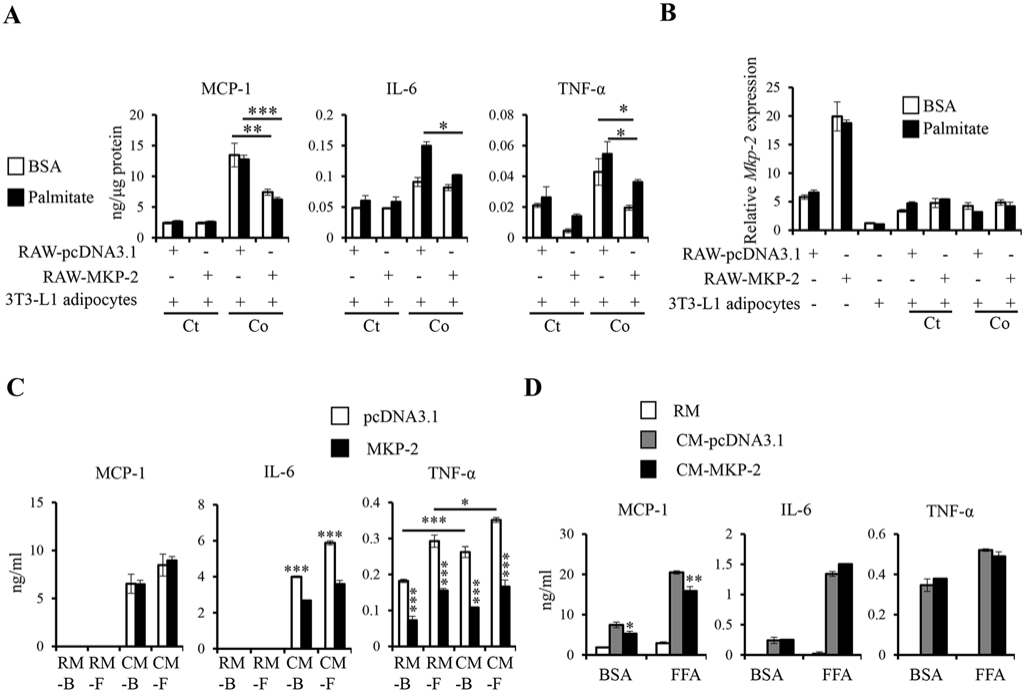
Overexpression of MKP-2 in macrophages inhibits inflammatory cytokine production during macrophage-adipocyte interaction. *(A-B)* Control or MKP-2 overexpressing RAW264.7 (1x10^5^) and differentiated 3T3-L1 cells were cultured either alone or together and stimulated with or without 750μM palmitate overnight. IL-6, TNF-α, and MCP-1 levels in the supernatants were determined by ELISA. Cytokine production was normalized to total protein of the cell lysates. mRNA of each sample was harvested to examine *Mkp-2* expression by qRT-PCR. Ct, control; Co, co-culture. *(C)* Control and MKP-2 overexpressing RAW264.7 cells were treated with conditioned medium (CM) derived from 3T3-L1 adipocytes treated with BSA (CM-B) or 750μM FFA (CM-F) for 24 h. Regular media containing BSA (RM-B) or 750μM FFA (RM-F) were used as control. Supernatants were harvested for ELISA analysis. *(D)* CM derived from vector transfected or MKP-2 overexpressing RAW264.7 treated with BSA or 750μM FFA were used to treat 3T3-L1 adipocytes for 24 h. Regular media containing BSA or 750μM FFA were used as control. Supernatants were harvested for ELISA analysis. RM, regular media; CM-pcDNA3.1, conditioned media derived from pcDNA3.1-transfected RAW264.7; CM-MKP-2, conditioned media derived from MKP-2 transfected RAW264.7. Data shown are representative of three independent experiments with similar results. Data are presented as mean ± SEM. *, p<0.05; **, p<0.01; ***, p<0.001.

Next, we performed *in vitro* macrophage-adipocyte co-culture experiment to further study the regulation of MKP-2 in inflammation during adipocyte-macrophage interaction. MKP-2 overexpressing macrophages were co-cultured with differentiated 3T3-L1 adipocytes for 24h either with or without palmitate stimulation. Interestingly, we observed significant increase in the levels of inflammatory cytokines, including IL-6, TNF-α, and chemokine MCP-1 in macrophage-adipocyte coculture without palmitate stimulation (Fig. 3A). FFA stimulation further increased the production of IL-6 and TNF-α, but not MCP-1. Importantly, overexpression of MKP-2 in macrophages greatly suppressed the production of inflammatory mediators, including IL-6, TNF-α, and MCP-1 with or without palmitate stimulation (Fig. 3A). Furthermore, *Mkp-2* was more highly expressed in RAW264.7 than 3T3-L1 adipocytes with or without palmitate stimulation (Fig. 3B). Even though the expression of MKP-2 was threefold higher in MKP-2 overexpressing RAW264.7 than pcDNA3.1-transfected RAW264.7, the expression of *Mkp-2* in different groups of co-culture cells was comparable but lower than pcDNA3.1-transfected RAW264.7 cells, which is likely due to the addition of the *MKp-2* lower adipocytes in the co-culture samples (Fig. 3B). Given that *Mkp-2* is highly expressed in macrophages, these adipocyte-macrophage interaction data provide evidence that overexpression of MKP-2 in macrophages suppresses the production of inflammatory cytokines during adipocyte-macrophage interaction.

To further investigate the regulatory role of MKP-2 in the initiation of inflammatory changes in both cells by production of secreted factors, we treated control or MKP-2 overexpressing RAW264.7 with CM derived from 3T3-L1 adipocytes treated with BSA or FFA for 24 h. It was observed that IL-6 and MCP-1 were not produced by RAW264.7 when treated with regular media (RM) with BSA or FFA (Fig. 3C). Interestingly, CM from 3T3-L1 adipocytes significantly increased IL-6, TNF-α, and MCP-1 production in macrophages (Fig. 3C). Importantly, overexpression of MKP-2 in RAW264.7 significantly reduced IL-6 and TNF-α but not MCP-1 production when cultured with CM derived from 3T3-L1 adipocytes (Fig. 3C). These data demonstrate that secreted factors from adipocytes trigger inflammatory cytokine expression in macrophages and MKP-2 expression in macrophages inhibits IL-6 and TNF-α production in macrophages induced by adipocyte-derived factors. Additionally, we also treated 3T3-L1 adipocytes with CM derived from RAW264.7 or MKP-2 overexpressing RAW264.7 treated with BSA or FFA. Interestingly, we found significant increase in IL-6, TNF-α, and MCP-1 levels after CM treatment (Fig. 3D). Importantly, only MCP-1 production was significantly lower in adipocytes when treated with CM derived from MKP-2 overexpressing RAW264.7 compared with cells treated with CM derived from control RAW264.7 (Fig. 3D). Thus, different secreted factors from macrophages are responsible for MCP-1 or IL-6/TNF-a expression by adipocytes and MKP-2 expression in macrophage inhibited the expression of factors that induce MCP-1 production in adipocytes. Taken together, the data suggest that MKP-2 expression in macrophages negatively regulates the production of IL-6, TNF-α and alters the expression of factors inducing MCP-1 production from adipocytes during adipocyte-macrophage interaction.

### MKP-2 expression in macrophages inhibits macrophage M1 activation

**Fig 4:**
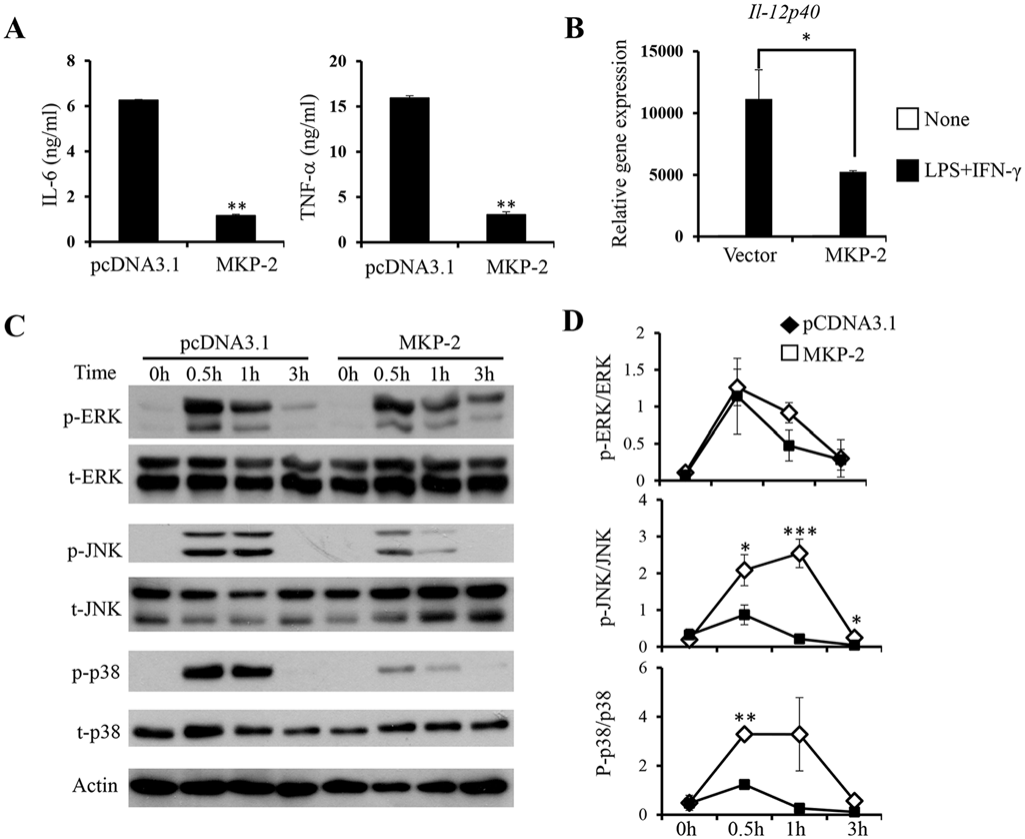
Overexpression of MKP-2 inhibits macrophage M1 activation and JNK/p38 activation. Control and MKP-2 overexpressing RAW264.7 cells were primed with 20 ng/mL IFN-γ for 12h followed by stimulation with 100 ng/mL LPS for another 12h. Culture supernatants were harvested for ELISA to detect IL-6 and TNF-α production *(A)*, and total RNA was extracted for qRT-PCR of *Il-12p40 (B)*. Cell lysates were harvested at 0 hour (h), 0.5h, 1h and 3h after M1 activator stimulation to determine the expression and activation of ERK, JNK, and p38 by western blot analysis *(C)*. *(D)* The intensity of phospho (p)-ERK, p-JNK, and p-p38 bands on the blots were quantified by ImageJ showing mean ± SEM from three independent experiments normalized to total ERK, JNK, and p38. Data are presented as mean ± SEM. *, p<0.05; **, p<0.01; ***, p<0.001.

Obesity is accompanied by macrophage infiltration into adipose tissue and a transition of ATMs from M2 to M1 activation status [7,26]. M1 macrophages accumulated in adipose tissue produce various inflammatory mediators such as IL-6 and TNF-α and promote the development of insulin resistance [8,11]. M2 macrophages, on the other hand, are anti-inflammatory and important for maintaining metabolic homeostasis in WAT [27]. To examine the function of MKP-2 in ATM activation, control and MKP-2 overexpressing macrophages were stimulated with IFN-γ for 12h followed by stimulation with LPS to induce macrophage M1 activation [28]. We found that MKP-2 overexpressing macrophages produced significantly reduced amount of IL-6 and TNF-α compared with control cells (Fig. 4A). The expression of *Il-12p40*, one of the M1 markers, was also significantly decreased in MKP-2 overexpressing cells (Fig. 4B). These results demonstrate that MKP-2 inhibits macrophage M1 activation. To examine the specificity of MKP-2 in macrophages for MAPK in response to M1 activation, the expression and activation of ERK, JNK, and p38 were determined. We found that the activation of all the three groups of MAPK was induced at 30 min and weaned off at 3 h after M1 stimulation (Fig. 4C and D). Overexpression of MKP-2 significantly inhibited the activation of JNK and p38, but not ERK, upon M1 activation (Fig. 4C and D). Taken together, the results suggest that MKP-2 may inhibit macrophage M1 activation by inhibiting JNK and p38 activation.

### MKP-2 expression in macrophages enhances macrophage M2 activation

**Fig 5:**
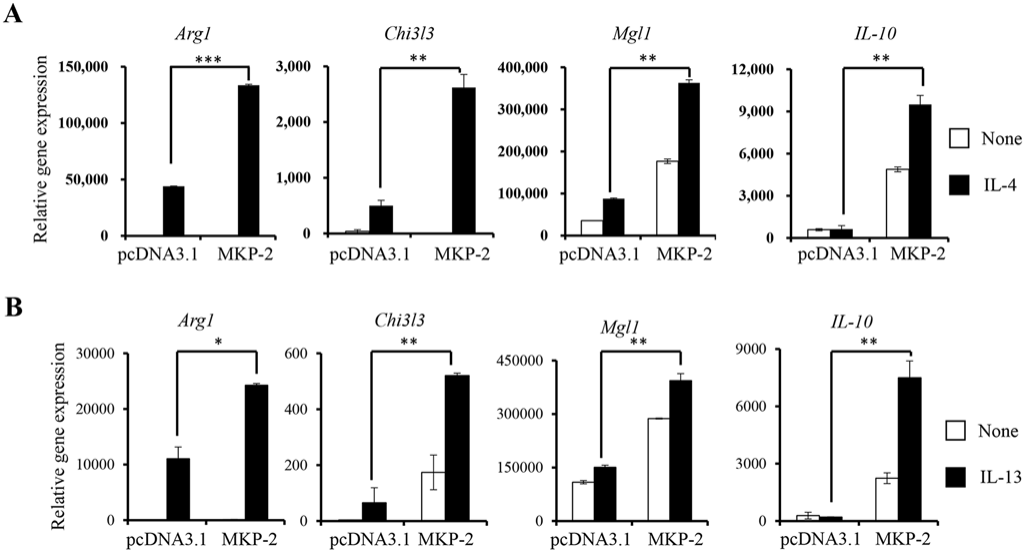
Overexpression of MKP-2 enhances macrophage M2 activation. *(A)* Control and MKP-2 overexpressing RAW264.7 cells were stimulated with 20 ng/mL IL-4 for 12h and total RNA was extracted to analyze the expression of M2 markers by qRT-PCR. *(B)* Control and MKP-2 overexpressing RAW264.7 cells were stimulated with 20 ng/mL IL-13 for 12h and total RNA was extracted to analyze expression of M2 markers by qRT-PCR. The data shown are representative of three independent experiments with similar results. Data are presented as mean ± SEM. *, p<0.05; **, p<0.01; ***, p<0.001.

Next, we examined if MKP-2 plays a role in macrophage M2 activation. We stimulated MKP-2 overexpressing or control cells with IL-4 to induce M2 activation. The expression of M2 markers, including *Arg1*, *Chi3l3*, *Mgl1*, and *Il-10* was found to be significantly increased in MKP-2 overexpressing cells compared with that in control cells (Fig. 5A). Furthermore, we also found that overexpression of MKP-2 increased the expression of M2 markers in response to IL-13, another factor known to induce macrophage M2 activation (Fig. 5B). Thus, these results demonstrate that MKP-2 modulates macrophage phenotypic switch by inhibiting M1 activation and promoting M2 activation.

### Differential regulation of macrophage M1 and M2 activation by JNK and p38

**Fig 6:**
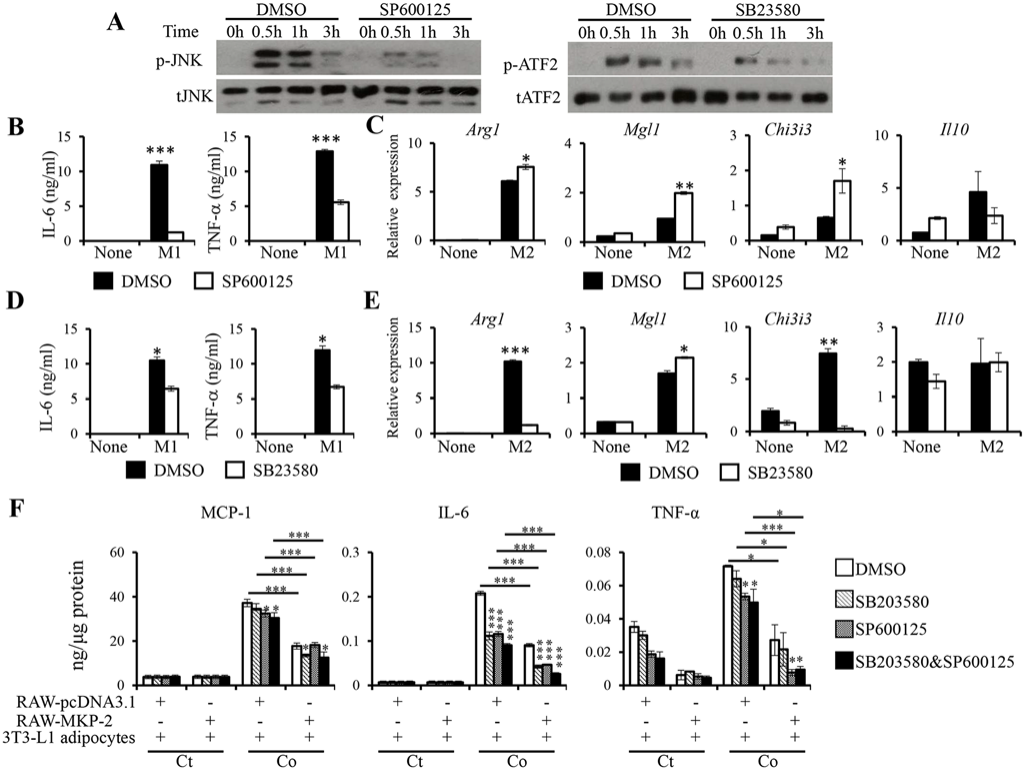
Inhibition of JNK and p38 in macrophages suppressed inflammatory cytokine expression in macrophages and in macrophage-adipocyte interaction. *(A)* RAW264.7 cells were stimulated with IFN-γ plus LPS in the presence of JNK-specific inhibitor SP600125 (20μM) (Sigma) or p38-specific inhibitor SB23580 (20μM) for the indicated time points. DMSO was used as vehicle control. The expression and activation of JNK or ATF2 were determined by western blot analysis. RAW264.7 cells were pretreated with SP600125 (20μM) followed by stimulation with M1 *(B)* or M2 *(C)* activation condition. The expression of M1 or M2 markers was determined. RAW264.7 cells were pretreated with SB23580 (20μM) followed by stimulation with M1 *(D)* or M2 *(E)* activation condition. The expression of M1 or M2 genes was analyzed. *(F)* Control and MKP-2 overexpressing RAW264.7 cells were pretreated with SP600125 (20μM) or SB23580 (20μM) or both for 1h before co-culture with differentiated 3T3-L1 adipocytes for 24h. The concentrations of MCP-1, IL-6, and TNF-α in culture supernatants were determined by ELISA. Cytokines production was normalized to total protein of the cell lysates. Ct, control; Co, co-culture. Data shown are representative of three independent experiments with similar results. Data are presented as mean ± SEM. *, p<0.05; **, p<0.01; ***, p<0.001.

Our study demonstrate that MKP-2 plays important roles in macrophage M1/M2 activation and inflammation during macrophage-adipocyte interaction possibly through JNK and/or p38. Next, we treated macrophages with JNK- or p38-specific inhibitor to examine their regulatory roles in macrophage M1 and M2 activation. As shown in Fig. 6A, treatment of macrophages with 20μM JNK-specific inhibitor, SP600125, greatly inhibited JNK activation in response to M1 stimulation, whereas treatment with 20μM p38-specific inhibitor, SB23580, reduced the phosphorylation of ATF2, a major substrate of p38. Consistent with previous findings, JNK inhibition resulted in reduced IL-6 and TNF-α production by macrophages in response to M1 activation (Fig. 6B). On the other hand, the expression of M2 markers, including *Arg1*, *Mgl1*, and *Chi3i3*, was significantly increased in macrophages upon M2 activation following JNK inhibition (Fig. 6C). Therefore, JNK activation promotes macrophage M1 activation and inhibits M2 activation. Similarly, inhibition of p38 significantly reduced the production of IL-6 and TNF-α by macrophages in response to M1 activation (Fig. 6D). The expression of M2 genes, including *Arg1* and *Chi3i3*, appears to require p38, as macrophages treated with p38-specific inhibitor expressed significantly lower levels of *Arg1* and *Chi3i3* (Fig. 6E). Interestingly, unlike *Arg1* and *Chi3i3*, the expression of *Mgl1* was significantly increased in response to M2 activation upon p38 inhibition.

To understand the regulation of macrophage-adipocyte interaction by JNK and p38, control RAW264.7 and MKP-2 overexpressing RAW264.7 were pretreated with JNK- or p38-specific inhibitor before being co-cultured with adipocytes. As shown in Fig. 6F, inhibition of JNK resulted in significantly reduced IL-6 and TNF-α production during the interaction of pcDNA3.1-transfected RAW264.7 with adipocytes, whereas p38 inhibition resulted in significantly reduced IL-6, but not MCP-1 and TNF-α following macrophage-adipocyte interaction (Fig. 6F). We also observed that inhibiting both JNK and p38 significantly reduced the production of all three cytokines during pcDNA3.1-transfected RAW264.7 co-cultured with adipocytes (Fig. 6F). Consistently, MKP-2 overexpression in RAW264.7 cells resulted in significantly reduced expression of all the three cytokines when co-cultured with adipocytes (Fig. 3A and 6F). p38 inhibition in MKP-2 overexpressing macrophages resulted in further reduction in MCP-1 and IL-6 production, while JNK inhibition led to further reduced IL-6 and TNFα production (Fig. 6F). Interestingly, only IL-6 production was further reduced upon the inhibition of both JNK and p38 inhibition in MKP-2 overexpressing RAW264.7 cells. These data suggest that JNK and p38 activation in macrophages differentially regulate cytokine expression and that MKP-2 potentially targets JNK and p38 to inhibit inflammatory cytokine production during adipocyte-macrophage interaction.

## Discussion

Obesity associated with chronic inflammation in adipose tissue is critical for the development of insulin resistance and other obesity-associated metabolic disorders [29]. MAPK such as JNK and p38 are essential regulators of inflammation. MKPs control the magnitude and duration of MAPK activation to determine the MAPK-regulated biological outcomes. Several MKPs have been shown to play specific roles in inflammation [30,31]. However, regulatory functions of MKPs in chronic inflammation in adipose tissue have not been well understood. In this study, we showed that MKP-2 plays an inhibitory role in inflammatory cytokine expression during adipocyte-macrophage interaction, suggesting the involvement of this protein in obesity-associated inflammation and metabolic disorders.

In obesity, the expanding adipose tissue releases a large numbers of mediators, including TNF-α, IL-6, and MCP-1 as well as FFA, which can interfere with insulin signaling [3]. FFA is considered as a primary link between obesity and inflammation. Plasma FFA level is elevated in obesity and diabetes in both humans and animals, and lowering FFA leads to increased insulin sensitivity [3]. ATMs, a major source of inflammatory mediators such as TNF-α and IL-6 [4], play critical role in WAT inflammation and in the development of insulin resistance [32]. MKP-2 was identified as a molecule encoded by an immediate-early gene [33]. We found that FFA increased both mRNA and protein expression of MKP-2 in macrophages around 3h after stimulation (Fig. 1B-C). Interestingly, in mouse WAT, MKP-2 is highly expressed in SVCs than in adipocytes and HFD decreased its expression in WAT SVCs (Fig. 1A), indicating that chronic nutrition stress changes the expression of this molecule in leukocytes in the WAT and a possible role of MKP-2 in WAT inflammation.

The development of adipose tissue inflammation and insulin resistance in obesity is associated with dysregulation of inflammatory signaling pathways including the MAP kinase pathways in adipose tissue. For instance, JNK activation is abnormally increased in adipose tissue in obesity [34]. Adipose tissue macrophage infiltration and activation is critical for the development of adipose tissue inflammation and insulin resistance. Proper regulation of inflammatory signaling pathway such as JNK in adipose tissue macrophages is important for maintaining adipose tissue homeostasis. For instance, it has been shown that macrophage-specific deletion of JNK in mice resulted in impaired macrophage M1 activation, reduced WAT macrophage infiltration and inflammation and improved insulin sensitivity in response to HFD [35]. Recently, it hase been shown that the PB1 domain-containing adaptor NBR1 functions as an organizer of MEKK3/MKK4, the activators of JNK, in macrophages to regulate JNK activation, ATM M1 activation and adipsoe tissue inflammation in obesity [36]. Myeloid-specific deletion of NBR1 resulted in impaired JNK activation associated with impaired macrophage M1 activation, reduced WAT macrophage infiltration and inflammation, and improved insulin sensitivity in response to chronic HFD exposure. It is likely that chronic exposure to HFD changes the expression of components of JNK and other inflammatory signaling pathways, which lead to adipose tissue inflammation and insulin resistance. The reduced expression of MKP-2 in SVCs is possibly a part of such changes which requires further investigation.

TNF-α is known as a major inflammatory mediator, which induces insulin resistance in adipose tissue [37]. Here, we discovered that overexpression of MKP-2 inhibits JNK activation and TNF-α production in macrophages in response to FFA stimulation (Figs. 2B-D). We further found that MKP-2 inhibited macrophage M1 activation through both JNK and p38 (Fig. 4C and D). Using JNK- and p38-specific inhibitors, we found that the expression of IL-6 and TNF-α by M1 macrophages requires both JNK and p38 (Fig. 6B and D). These data demonstrate that JNK and p38 promote macrophage M1 activation, and MKP-2 potentially targets both JNK and p38 to inhibit M1 macrophage activation.

Till date, there exist several controversies on the substrates of MKP-2. MKP-2 has been shown to inactivate ERK in B cells and mouse embryonic fibroblasts [38,39]. Al-Mutairi et al showed that in macrophages MKP-2 inhibits the activation of JNK and p38, but not ERK [16], whereas Cornell et al demonstrated that in macrophages MKP-2 is an ERK phosphatase [15]. Our observations of decreased JNK and p38 activation and reduced expression of inflammatory cytokines in MKP-2 overexpressing macrophages upon M1 activation support that it is a negative regulator of both JNK and p38.

Interestingly, we found that in addition to the suppression of macrophage M1 activation, MKP-2 increases macrophage M2 activation (Fig. 5A and B). We examined the expression of molecules important for M2 activation, including IRF4, PPAR-γ and the phosphorylation of STAT6. Our results show that MKP-2 does not regulate their expression or activation (S1 Fig.). MAPK activation in response to M2 culture condition was not detectable. However, it is possible that MKP-2 promotes macrophage M2 activation through the inhibition of JNK and p38, as we observed that inhibition of JNK and p38 increased the expression of M2 marker (Fig. 6C and E). Currently, we are investigating the mechanism by which MKP-2 regulates macrophage M2 activation.

Using macrophage-adipocyte co-culture system, we investigated the role of MKP-2 in inflammation during the interaction of the two major cell types in adipose tissue. ATMs are thought to be dominant contributors to the development of obesity-associated inflammation [32]. It is proposed that there is a paracrine loop involving adipocyte-derived FFAs and macrophage-derived TNF-α establishing a vicious cycle that aggravates inflammatory changes in obese adipose tissue [40]. Interestingly, we observed increased expression of inflammatory cytokines, including IL-6, TNF-α, and MCP-1 upon macrophage-adipocyte interaction without FFA stimulation (Fig. 3A), suggesting that the interaction between adipocytes and macrophages could induce the expression of inflammatory cytokines thereby contributing to the development of adipose tissue inflammation. The expression of MCP-1 is not enhanced by FFA stimulation, suggesting that macrophage infiltration itself could lead to monocyte/macrophage recruitment into adipose tissue to augment inflammation. MKP-2 expression in macrophages significantly suppressed the expression of these cytokines with or without FFA stimulation, demonstrating that macrophages play a major role in inflammation triggered by macrophage-adipocyte interaction and MKP-2 inhibits such inflammation. Interestingly, we observed significantly decreased IL-6 and TNF-α production in MKP-2 overexpressing RAW264.7 when treated with CM derived from adipocytes (Fig. 3C). CM derived from MKP-2 overexpressing RAW264.7 inhibited MCP-1 production in adipocytes (Fig. 3D), suggesting that MKP-2 expression in macrophages inhibits the expression of secreted factors that important for MCP-1 production during adipocyte-macrophage interaction. In addition, when macrophages were pretreated with JNK inhibitor, p38 inhibitor, or JNK/p38 inhibitors before being co-cultured with adipocytes, significant decreases in IL-6, TNF-α, and MCP-1 levels were observed during adipocyte-macrophage interaction (Fig. 6F), suggesting that both JNK and p38 activation in macrophages are important for inflammation during adipocyte-macrophage interaction. However, owing to the complexity of adipose tissue, which harbors various types of cells such as mature adipocytes, preadipocytes, fibroblasts, and immune cells, it is difficult to completely mimic the *in vivo* environment in adipose tissue by co-culturing adipocytes with macrophages. Additionally, adipocytes are able to secrete proinflammatory mediators such as IL-6, TNF-α, and MCP-1 [41], which could be regulated by MKP-2. This necessitates further studies to verify the function of MKP-2 in adipose tissue inflammation and obesity-associated insulin resistance.

In summary, our study demonstrates that MKP-2 expression in macrophages negatively regulates macrophage M1 activation and inflammation during adipocyte-macrophage interaction by inhibiting JNK and p38 activation. Further understanding the mechanisms by which MKP-2 regulates adipose tissue inflammation will shed new lights on the regulation of obesity-associated inflammation and metabolic disorders, including insulin resistance and T2D.

## Supporting Information

S1 Fig Overexpression of MKP-2 in macrophages does not change the expression and activation of M2 regulators. (A) Control and MKP-2 overexpressing RAW264.7 cells were stimulated with 20 ng/mL IL-4 for the indicated time points to examine the expression of PPARγ or the phosphorylation of STAT6 by western blot analysis. (b) IRF4 expression in response to IL-4 stimulation was determined by quantitative real-time PCR (qRT-PCR). Data shown are representative of three independent experiments with similar results. Data are presented as mean ± SEM. *, p<0.05; **, p<0.01; ***, p<0.001.
(TIF)
