# Supplementary figures and images for: MAP Kinase Phosphatase 2 Regulates Macrophage-Adipocyte Interaction
Source: PLoS One. 2015 Mar 27;10(3):e0120755. doi: 10.1371/journal.pone.0120755 (PMC4376689; doi:10.1371/journal.pone.0120755)

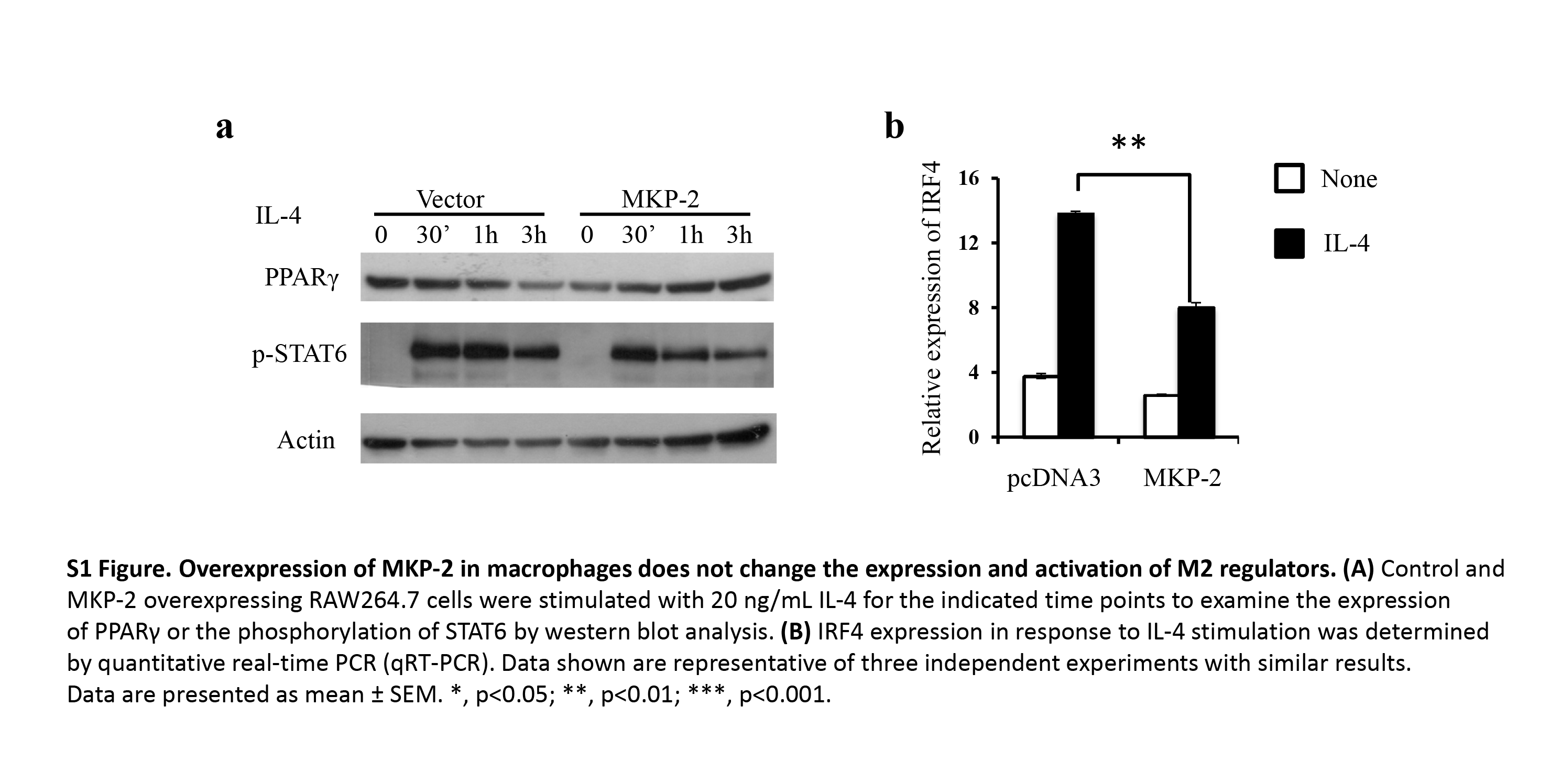

Supplement: S1 Fig — (A) Control and MKP-2 overexpressing RAW264.7 cells were stimulated with 20 ng/mL IL-4 for the indicated time points to examine the expression of PPARγ or the phosphorylation of STAT6 by western blot analysis. (b) IRF4 expression in response to IL-4 stimulation was determined by quantitative real-time PCR (qRT-PCR). Data shown are representative of three independent experiments with similar results. Data are presented as mean ± SEM. *, p<0.05; **, p<0.01; ***, p<0.001. (TIF) [file pone.0120755.s001.tif]
